# Whole-body MRI in oncology: can a single anatomic T2 Dixon sequence replace the combination of T1 and STIR sequences to detect skeletal metastasis and myeloma?

**DOI:** 10.1007/s00330-022-09007-8

**Published:** 2022-08-04

**Authors:** Ophelye Chiabai, Sandy Van Nieuwenhove, Marie-Christiane Vekemans, Bertrand Tombal, Frank Peeters, Joris Wuts, Perrine Triqueneaux, Patrick Omoumi, Thomas Kirchgesner, Nicolas Michoux, Frédéric E. Lecouvet

**Affiliations:** 1 Department of Radiology and Medical Imaging, Cliniques Universitaires Saint Luc, Institut de Recherche Expérimentale & Clinique (IREC), Université Catholique de Louvain (UCLouvain), Hippocrate Avenue, 10, B-1200 Brussels, Belgium; 2 Department of Internal Medicine, Hematology Unit, Cliniques Universitaires Saint Luc, Institut de Recherche Expérimentale & Clinique (IREC), Université Catholique de Louvain (UCLouvain), Brussels, Belgium; 3 Department of Surgery, Urology Unit, Cliniques Universitaires Saint Luc, Institut de Recherche Expérimentale & Clinique (IREC), Université Catholique de Louvain (UCLouvain), Brussels, Belgium; 4 Department of Electronics and Informatics (ETRO), Vrije Universiteit Brussel, Brussels, Belgium; 5 Department of Radiology, Centre Hospitalier Universitaire Vaudois (CHUV), Lausanne, Switzerland

**Keywords:** Magnetic resonance imaging, Whole-body imaging, Cancer, Metastasis, Multiple myeloma

## Abstract

**Objectives:**

To compare the diagnostic accuracy of a single T2 Dixon sequence to the combination T1+STIR as anatomical sequences used for detecting tumoral bone marrow lesions in whole-body MRI (WB-MRI) examinations.

**Methods:**

Between January 2019 and January 2020, seventy-two consecutive patients (55 men, 17 women, median age = 66 years) with solid (prostate, breast, neuroendocrine) cancers at high risk of metastasis or proven multiple myeloma (MM) prospectively underwent a WB-MRI examination including coronal T1, STIR, T2 Dixon and axial diffusion-weighted imaging sequences. Two radiologists independently assessed the combination of T1+STIR sequences and the fat+water reconstructions from the T2 Dixon sequence. The reference standard was established by consensus reading of WB-MRI and concurrent imaging available at baseline and at 6 months. Repeatability and reproducibility of MRI scores (presence and semi-quantitative count of lesions), image quality (SNR: signal-to-noise, CNR: contrast-to-noise, CRR: contrast-to-reference ratios), and diagnostic characteristics (Se: sensitivity, Sp: specificity, Acc: accuracy) were assessed *per*-skeletal region and *per*-patient.

**Results:**

Repeatability and reproducibility were at least good regardless of the score, region, and protocol (0.67 ≤ AC1 ≤ 0.98). CRR was higher on T2 Dixon fat compared to T1 (*p* < 0.0001) and on T2 Dixon water compared to STIR (*p* = 0.0128). In the *per*-patient analysis, Acc of the T2 Dixon fat+water was higher than that of T1+STIR for the senior reader (Acc = +0.027 [+0.025; +0.029], *p* < 0.0001) and lower for the junior reader (Acc = −0.029 [−0.031; −0.027], *p* < 0.0001).

**Conclusions:**

A single T2 Dixon sequence with fat+water reconstructions offers similar reproducibility and diagnostic accuracy as the recommended combination of T1+STIR sequences and can be used for skeletal screening in oncology, allowing significant time-saving.

**Key Points:**

*• Replacement of the standard anatomic T1 + STIR WB-MRI protocol by a single T2 Dixon sequence drastically shortens the examination time without loss of diagnostic accuracy.*

*• A protocol based on fat + water reconstructions from a single T2 Dixon sequence offers similar inter-reader agreement and a higher contrast-to-reference ratio for detecting lesions compared to the standard T1 + STIR protocol.*

*• Differences in the accuracy between the two protocols are marginal (+ 3% in favor of the T2 Dixon with the senior reader; −3% against the T2 Dixon with the junior reader).*

**Supplementary Information:**

The online version contains supplementary material available at 10.1007/s00330-022-09007-8.

## Introduction

Over recent years, whole-body magnetic resonance imaging (WB-MRI) has demonstrated a high diagnostic performance and is now recommended in clinical guidelines for skeletal lesion detection and follow-up in patients with metastases from solid cancers and multiple myeloma (MM) [1–6]. Standard WB-MRI examinations combine anatomic T1 and short-Tau inversion recovery (STIR) sequences and functional diffusion-weighted imaging (DWI) sequences [7–9]. T1 is the reference sequence for marrow lesion detection and characterization; STIR increases the sensitivity for lesion detection [10, 11]. DWI sequences add diagnostic value to anatomic sequences thanks to a high lesion to background contrast and extend cancer screening to lymph nodes and extraskeletal organs [12–16]. A limitation of WB-MRI is its duration and various initiatives are undertaken to accelerate the different sequences [17–19].

The Dixon technique relies on the chemical shift between protons of water and fat and decomposes the signal from these two components in the same voxel. A Dixon sequence generates four types of images: in-phase (IP) (equivalent to non-fat-suppressed anatomic images), out-of-phase (OP), water images (equivalent to fat-suppressed), and fat images (equivalent to water-suppressed) [20–22]. A single T2 Dixon sequence combines STIR-like information thanks to water images, and T1-like information thanks to fat images [23–25]. The diagnostic performance of the T2 Dixon sequence for detecting metastatic and MM lesions and its ability to replace the T1+STIR sequences has been demonstrated in spinal MRI examinations [26–28].

Herein, we hypothesized that a faster WB-MRI protocol using a single T2 Dixon sequence may be used without loss of diagnostic accuracy for detecting neoplastic bone marrow lesions. This study compares the (i) repeatability and reproducibility, (ii) image quality (signal-to-noise ratio (SNR); contrast-to-noise ratio (CNR); contrast-to-reference ratio (CRR), and (iii) diagnostic accuracy of combined fat + water reconstructions from a single T2 Dixon sequence with those of the combined T1+STIR sequences.

## Materials and methods

### Patient population (Fig. 1)

The study included consecutive adult (≥ 18 years old) patients with either solid cancers at high risk for metastases or biopsy-proven newly diagnosed multiple myeloma (MM).

**Fig. 1:**
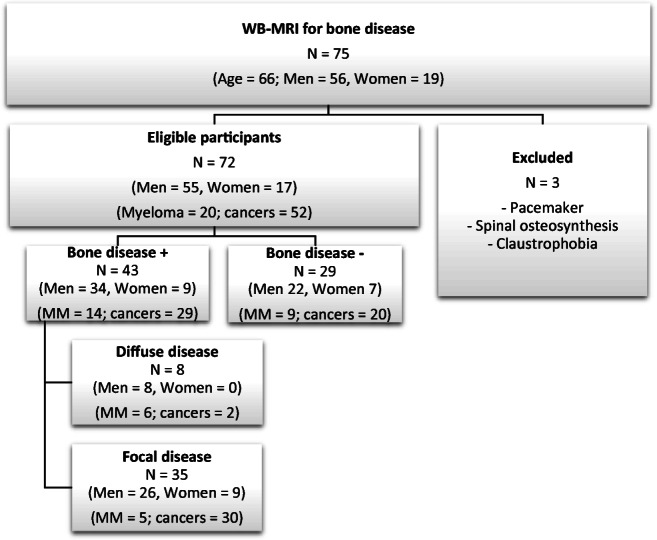
Patient demographics

The indication for WB-MRI in newly diagnosed patients with solid cancers was validated in multidisciplinary tumor boards using cancer-specific indications.

In prostate cancer, high risk for metastasis was defined according to the National Comprehensive Cancer Network (NCCN) guidelines: cancer with ≥ 20 ng/ml prostate-specific antigen, Gleason score ≥ 8, and Union for International Cancer Control clinical T stage 3 or 4 [29]. In breast cancer, high risk was defined according to the European Society of Medical Oncology (ESMO) criteria: clinically positive axillary nodes, tumors > 5 cm, aggressive biology and clinical signs, and symptoms or laboratory values suggesting the presence of metastases [30]. In neuroendocrine cancer, high risk for metastasis was defined as follows: tumor grade ≥ III, tumor size ≥ 3 cm in diameter, T4 stage, N1 stage, histology of neuroendocrine carcinoma, and esophagus as the primary site [31].

Exclusion criteria were previous history of treated cancer, patients with more than one primary cancer, and contraindications to MRI.

All patients underwent a WB-MRI examination for the assessment of bone involvement from January 2019 to January 2020. This single-center study was approved by the institutional ethics committee. No informed consent was required for the retrospective reading of prospectively acquired data.

### MRI protocol

All acquisitions were performed on a 3.0-T magnet (Ingenia, Philips Healthcare). Patients were imaged head first in the supine position, from head to proximal femurs, covered with head, neck, spine, and two 6-element body matrix coils. 3DT1, STIR, T2 Dixon and DWI sequences were performed. Synthetic water, fat, IP, and OP images were automatically reconstructed from the Dixon acquisition. After acquiring three stacks of T1, STIR, and T2 Dixon images in the coronal plane and four stacks of DWI in the axial plane, a single stack of pasted reformatted coronal images was constructed for each sequence. The total acquisition time was 49 min. Imaging parameters are detailed in Table 1.

**Table 1:** Imaging parameters

Parameters	3D T1 FSE	T2 Dixon	STIR	DWI
Scan orientation	Coronal	Coronal	Coronal	Axial
Phase-encoding direction	feet-head	Left-right	Feet-head	Anterior-posterior
Acquired voxel size (mm) (read x phase x slice)	1.14 × 1.30 × 1.20	1.19 × 1.40 × 4	1.2 × 1.60 × 4	4.4 × 4.8 × 6
Field of view (mm) (read x phase x slice)	500 × 350 × 252	350 × 496 × 250	500 × 350 × 256	440 × 348 × 305
No. of stations	3	3	3	4
Overlap between stations (mm)	50	50	50	50
Coverage in z-axis (mm)	1050	1050	1050	1070
Phase oversampling (mm)	200 × 200	No	180×120	0
TR/TE (ms)	285 / 21	3722 / 55	7500 / 50	6000 / 59
TI (ms)	-	-	200	250
No. of signals acquired	2	1	2	1
Turbo factor	50	11	30	NA
Bandwidth (Hz)	1052	763	640	107
Fat suppression technique	_	Dixon	STIR	SPAIR
*b* values, s/mm^2^	-	-	-	0-50-150-1000
Acquisition time, per stack	3 min 38 s	4 min 13 s	3 min 39 s	3 min 36 s
Total acquisition time, per sequence	3 × 3 min 38 s	3 × 4 min 13 s	3 × 3min 39 s	4 × 3 min 36 s
	10 min 54 s	12 min 39 s	10 min 57 s	14 min 24 s

*Notes. FSE*, fast spin-echo; *STIR*, short-TI inversion recovery sequence; *DWI,* diffusion-weighted imaging sequence; *TR,* repetition time; *TE*, echo time; *TI*, inversion time

### MRI readings

All images were stored and read on the institutional Picture Archiving and Communication System (Carestream Vue). The combinations of T1+STIR sequences and of fat+water reconstructions from the T2 Dixon sequence were assessed by two radiologists with 2- and 15-years’experience in WB-MRI. Readings were performed independently, randomly, and blinded to clinical information and to DWI. Images were assessed twice by the junior reader at a 3-month interval for measuring the repeatability. IP and OP images derived from the Dixon acquisition were not considered for analysis as they did not add diagnostic value for lesion detection based on preliminary evaluation and literature [26]. DWI was used during the consensus session for determining the reference standard.

### Bone involvement

Four patterns of bone marrow involvement were considered, as previously described: the normal, focal, diffuse, and “salt-and-pepper” patterns [6, 12, 32–36].

Normal marrow was defined as showing a homogeneous high signal intensity on T1 and fat images, and homogeneous low signal intensity on STIR and water images. A focal bone marrow lesion (focal metastasis in solid cancers or focal plasmocytoma in MM, with a minimal diameter of 5 mm) was defined as a low signal intensity area on T1 and fat images (similar to or lower than the signal intensity of discs and muscles on T1, very low signal on fat), intermediate to high signal intensity on STIR and water images, and high signal intensity on high *b*-value DW images. Diffuse marrow infiltration (diffuse metastatic disease in solid cancers and diffuse bone involvement in MM) was defined as homogeneous low signal intensity of the bone marrow on T1 and fat images (similar to or lower than the signal intensity of discs and muscles on T1, very low signal on fat), an intermediate to high signal intensity of the marrow on STIR and water images, and high signal intensity of the marrow on high *b*-value images. The fourth “salt-and-pepper” pattern of infiltration was observed in MM, defined by the presence of innumerable unmeasurable tiny foci with low signal intensity on T1 and fat and intermediate to high signal intensity on STIR and water images.

Eight skeletal regions were studied: skull, thoracic cage, cervical, thoracic, lumbar spine, pelvis, humerus, and femurs. In the *per*-region analysis, two scores were assessed: a categorical score (presence of lesion = yes/no) and a semi-quantitative score corresponding to the count of lesions (0 = no lesion; 1 = 1 to 5 lesions; 2 = 6 to 10 lesions; 3 = more than10 lesions; 4 = diffuse disease). In the *per*-patient analysis, two similar scores were assessed: a total categorical score (patient positive if at least one positive region = yes/no) and a total semi-quantitative score corresponding to the total count of lesions in all regions considered (0 = no lesion; 1 = 1 to 5 lesions; 2 = 6 to 10 lesions; 3 = more than10 lesions; 4 = diffuse disease).

### Reference standard and adjudication of readings

In the absence of a systematic pathologic gold standard, a best valuable comparator (BVC) was used as the reference standard for tumoral bone marrow involvement. This BVC was constructed during a consensus session by the readers along with a third reader (radiologist with 15 years’experience in WB-MRI) and clinicians, relying on the concurrent study of all baseline WB-MRI sequences (T1, STIR, T2 fat and water, DWI), clinical data, and other available imaging studies [37, 38]. At least one systematic 6-month follow-up WB-MRI examination was performed in all patients. A radiographic skeletal survey was performed at diagnosis and repeated after 6 months in all MM patients. In solid cancer patients, baseline and follow-up evaluations using other techniques (bone scintigraphy, thoraco-abdomino-pelvic (TAP) CT, positron-emission tomography (PET)-CT) were available, depending on the primary cancer. The causes of false positive (FP) / false negative (FN) were determined for each protocol and reader during the consensus session.

### Image quality

As the value of an imaging sequence used for lesion detection depends on the contrast between lesions and their environment, SNR (SNR=SI/σ^background^), CNR (CNR=(SI^lesion^–SI^reference^)/σ^background^), and CRR (CRR = (SI^lesion^–SI^reference^)/SI^reference^) were assessed (SI: mean signal intensity in the region of interest (ROI), σ^background^: standard deviation of SI in the image background). Measurements were performed by the junior reader after verification of the true pathologic nature of the measured lesions according to the reference standard. A single ROI, the largest possible, was drawn within bone lesions without including bone cortices, with a maximum of five lesions per patient and a minimal diameter of 10 mm. For spine lesions, the reference ROI was chosen in an unequivocally non-involved bone marrow area of the involved vertebra or in case of large lesions in the bone marrow of the closest uninvolved vertebra. For other bone lesions, the reference ROI was chosen in an unequivocally non-involved area. Two ROIs, the largest possible, were drawn in the image background to assess the noise. All measurements were performed on the same coronal slice.

### Statistical analysis

Due to the non-normality of data distributions (according to the Shapiro-Wilk test at *p* < 0.05), the comparison of SNR, CNR, and CRR measurements between protocols was performed using Wilcoxon’s signed-rank test [39].

Repeatability and reproducibility of MRI (categorical and semi-quantitative) scores were assessed using Gwet’s AC1 agreement coefficient [40]. Strength of intra-(repeatability) and inter-reader agreement (reproducibility) was interpreted according to the Landis-Koch’s scale: AC1 < 0.20 = poor; 0.21 ≤ AC1 < 0.40 = fair; 0.41 ≤ AC1 < 0.60 = moderate; 0.61 ≤ AC1 < 0.80 = good and AC1 ≥ 0.81 = very good [41].

Diagnostic characteristics and agreement between each protocol and the reference standard were assessed. True positives (TP), false positives (FP), false negatives (FN), true negatives (TN), sensitivity (Se), specificity (Sp), accuracy (Acc=(TP + TN)/(TP + FP + FN + TN)), and AC1 were reported for the *per*-region/*per*-patient analyses (for both readers). A two-sided exact test was used for comparing the proportion of lesions/patients correctly detected in the *per*-region/*per*-patient analyses by each protocol compared to the reference standard (significance level after Bonferroni-like correction *p* < 0.0083). This analysis was performed for the whole cohort of patients, and for two subgroups (*N* = 20 patients with MM and *N* = 52 patients with metastases; see Supplementary Materials).

Difference in Acc between protocols in the *per*-patient analysis was assessed using a resampling procedure (without replacement) based on 300 samples of *N* = 54 patients randomly drawn from the whole cohort of *N* = 72 patients (respectively based on samples of *N* = 15/*N* = 38 patients randomly drawn from the subgroups of patients with MM/metastases). A paired t-test was then performed from which the mean difference in Acc was reported for each of the three groups that were studied (significance level *p* < 0.0083; see Supplementary Materials).

Finally, an agreement between each protocol and the reference standard on the semi-quantitative score in the *per*-patient analysis was assessed using the AC1 coefficient.

All calculations were done with Statsdirect Statistical Software v3.3.5 and with Matlab R2021b.

## Results

### Patient characteristics

Seventy-two patients were included (55 men, 17 women, median age = 66 years [64 years; 68 years]). Twenty were examined for staging biopsy-proven MM. Fifty-two had newly diagnosed solid cancers at high risk for metastases (30 prostate cancers; 10 breast cancers; 12 neuroendocrine cancers) (Fig. 1). According to the reference standard, 35 (5 MM; 30 solid cancer patients) had focal bone lesions; 8 (6 MM; 2 solid cancer patients) had diffuse marrow involvement; and 29 had normal marrow (9 MM; 20 solid cancer patients). Due to the semi-quantitative scoring, the exact number of focal lesions cannot be provided. There were at least 3, 91, 26, 80, 64, 220, 8, and 27 focal lesions in the skull, thorax, cervical, dorsal, lumbar spine, pelvis, humerus, and femurs, respectively; in total, at least 519 tumoral bone marrow lesions were observed.

### Repeatability and reproducibility

In the *per*-region analysis, repeatability of readings was at least good regardless of the region, score (categorical or semi-quantitative), and protocol (T1+STIR or fat+water) (Table 2). Reproducibility was at least good regardless of the region, score, and protocol. In the *per*-patient analysis, considering the worst level of agreement measured in both analyses (intra- and inter-reader agreement), reproducibility was at least good regardless of both the score and protocol.

**Table 2:** Repeatability and reproducibility of MRI readings assessed using Gwet’s AC1 coefficient

	Reproducibility		Repeatability	
	Inter-reader agreement		Intra-reader agreement	
Categorical score = positive yes/no				
	**T1+STIR**	**Fat + water**	**T1+STIR**	**Fat + water**
Skull	0.98 [0.94; 1.02]	0.98 [0.94; 1.02]	0.96 [0.91; 1.01]	0.98 [0.94; 1.02]
Thorax	0.90 [0.80; 1.00]	0.95 [0.88; 1.02]	0.83 [0.70; 0.95]	0.83 [0.70; 0.95]
Cervical spine	0.92 [0.84; 1.00]	0.98 [0.94; 1.02]	0.88 [0.78; 0.98]	0.96 [0.91; 1.02]
Thoracic spine	0.87 [0.75; 0.98]	0.89 [0.79; 1.00]	0.87 [0.75; 0.94]	0.87 [0.75; 0.98]
Lumbar spine	0.76 [0.61; 0.91]	0.97 [0.92; 1.02]	0.76 [0.61; 0.91]	0.89 [0.79; 1.00]
Pelvis	0.84 [0.71; 0.96]	0.84 [0.72; 0.96]	0.67 [0.50; 0.84]	0.78 [0.64; 0.93]
Humerus	0.91 [0.83; 1.00]	0.94 [0.87; 1.01]	0.85 [0.74; 0.96]	0.92 [0.84; 1.00]
Femurs	0.88 [0.78; 0.98]	0.90 [0.81; 1.00]	0.78 [0.65; 0.92]	0.88 [0.77; 0.98]
*Per*-patient	0.82 [0.68; 0.95]	0.82 [0.69; 0.95]	0.74 [0.58; 0.89]	0.85 [0.72; 0.97]
Semi-quantitative score = count of lesions				
	**T1+STIR**	**Fat + water**	**T1+STIR**	**Fat + water**
Skull	0.98 [0.95; 1.02]	0.98 [0.95; 1.02]	0.97 [0.92; 1.01]	0.98 [0.95; 1.02]
Thorax	0.93 [0.87; 1.00]	0.97 [0.92; 1.01]	0.88 [0.80; 0.97]	0.88 [0.80; 0.97]
Cervical spine	0.92 [0.85; 0.99]	0.98 [0.95; 1.01]	0.91 [0.83; 0.98]	0.97 [0.93; 1.01]
Thoracic spine	0.90 [0.82; 0.98]	0.92 [0.85; 0.99]	0.91 [0.84; 0.99]	0.89 [0.81; 0.97]
Lumbar spine	0.82 [0.72; 0.92]	0.95 [0.90; 1.01]	0.82 [0.72; 0.92]	0.89 [0.81; 0.97]
Pelvis	0.85 [0.76; 0.94]	0.88 [0.80; 0.97]	0.77 [0.66; 0.88]	0.78 [0.68; 0.89]
Humerus	0.93 [0.86; 1.00]	0.95 [0.89; 1.01]	0.88 [0.80; 0.97]	0.93 [0.87; 1.00]
Femurs	0.92 [0.85; 0.99]	0.93 [0.87; 1.00]	0.82 [0.72; 0.92]	0.90 [0.82; 0.98]
*Per*-patient	0.76 [0.65; 0.87]	0.83 [0.73; 0.93]	0.71 [0.59; 0.83]	0.78 [0.67; 0.89]

### Image quality

T1 images had significantly higher SNR compared to T2 Dixon fat images for both tissue types (*p*^lesion^ < 0.0001; *p*^reference^ < 0.0093). T2 Dixon fat images had significantly higher CRR compared to T1 images (*p* < 0.0001), and T2 Dixon water images had a significantly higher CRR compared to STIR images (*p* = 0.0128) (Table 3).

**Table 3:** Image quality evaluation using SNR (signal-to-noise ratio), CNR (contrast-to-noise ratio), and CRR (contrast-to-reference ratio)

SNR					*p* value of the comparison	
	**T1**	**STIR**	**Fat Dixon**	**Water Dixon**	**T1 vs fat Dixon**	**STIR vs water Dixon**
Lesion	184 [136; 258]	144 [113; 252]	77.4 [57.9; 94.1]	142 [119; 202]	< 0.0001	0.3388
Reference	346 [268; 493]	98.5 [64.4; 188]	272 [207; 301]	71 [63; 96]	0.0093	0.0687
**CNR**					***p*** **value of the comparison**	
	**T1**	**STIR**	**Fat Dixon**	**Water Dixon**	**T1 vs fat Dixon**	**STIR vs water Dixon**
(Lesion - Reference)/σ	160 [117; 201]	73.8 [49.7; 96.6]	179 [135; 227]	72.1 [52.6; 98.7]	0.6876	0.8518
**CRR**					***p*** **value of the comparison**	
	**T1**	**STIR**	**Fat Dixon**	**Water Dixon**	**T1 vs fat Dixon**	**STIR vs water Dixon**
(Lesion - Reference)/reference	0.49 [0.39; 0.53]	0.78 [0.58; 0.97]	0.68 [0.65; 0.75]	0.95 [0.81; 1.12]	< 0.0001	0.0128

*Note*: σ= noise

SNR in the lesion and in the reference tissue, CNR (taking fat as reference), and CRR (ratio between lesion and reference tissue) measurements are provided. Median values and [95% confidence intervals] are reported. Significance threshold of the 2-sided Wilcoxon test: *p* < 0.05

### Diagnostic characteristics

In the *per*-region analysis, Se of T1+STIR was ≥ 93% for the senior reader (≥ 89% for the junior) regardless of the region (Table 4). Sp was ≥ 94% for the senior reader (≥ 82% for the junior) regardless of the region. Se of T2 Dixon fat+water was ≥ 93% for the senior reader (≥ 93% for the junior) regardless of the region. Sp was ≥ 91% for the senior reader (≥ 75% for the junior).

**Table 4:** Diagnostic characteristics and agreement between the protocols and the Reference Standard in the whole cohort of patients (*N* = 72)

	TP	FP	FN	TN	Se	Sp	Acc	AC1	Proportion	*p* value
									Difference (in %)	
Standard (3D T1 + STIR)										
Skull	**11**	**1**	**0**	**60**	**100 [72; 100]**	**98 [91; 100]**	**99 [93; 100]**	**0.98 [0.94; 1.02]**	**-**	**> 0.9999**
	11	0	0	61	100 [72; 100]	100 [94; 100]	100 [95; 100]	1.00 [1.00; 1.00]	-	> 0.9999
Thorax	**21**	**5**	**1**	**45**	**95 [77; 100]**	**90 [78; 97]**	**92 [83; 97]**	**0.85 [0.73; 0.97]**	**-**	**0.2188**
	21	1	1	49	95 [77; 100]	98 [89; 100]	97 [90; 100]	0.95 [0.88; 1.02]	-	> 0.9999
Cervical spine	**12**	**4**	**0**	**56**	**100 [74; 100]**	**93 [84; 98]**	**94 [86; 98]**	**0.92 [0.84; 1.00]**	**-**	**0.1250**
	12	0	0	60	100 [74; 100]	100 [94; 100]	100 [95; 100]	1.00 [1.00; 1.00]	-	> 0.9999
Thoracic spine	**25**	**5**	**1**	**41**	**96 [80; 100]**	**89 [76; 96]**	**92 [83; 97]**	**0.84 [0.72; 0.96]**	**-**	**0.2188**
	26	1	0	45	100 [87; 100]	98 [89; 100]	99 [93; 100]	0.97 [0.92; 1.02]	-	> 0.9999
Lumbar spine	**24**	**8**	**3**	**37**	**89 [71; 98]**	**82 [68; 92]**	**85 [74; 92]**	**0.70 [0.54; 0.87]**	**-**	**0.2266**
	26	1	1	44	96 [81; 100]	98 [88; 100]	97 [90; 100]	0.95 [0.88; 1.02]	-	> 0.9999
Pelvis	**38**	**5**	**2**	**27**	**95 [83; 99]**	**84 [67; 95]**	**90 [81; 96]**	**0.81 [0.68; 0.94]**	**-**	**0.4531**
	37	2	3	30	93 [80; 98]	94 [79; 99]	93 [85; 98]	0.86 [0.75; 0.98]	-	> 0.9999
Humeri	**13**	**6**	**0**	**53**	**100 [75; 100]**	**90 [79; 96]**	**92 [83; 97]**	**0.87 [0.77; 0.98]**	**+ 0.08 [+ 0.01; + 0.16]**	0.0313*
	13	2	0	57	100 [75; 100]	97 [88; 100]	97 [90; 100]	0.96 [0.90; 1.02]	-	0.5000
Femurs	**19**	**5**	**1**	**47**	**95 [75; 100]**	**90 [79; 97]**	**92 [83; 97]**	**0.86 [0.74; 0.97]**	**-**	**0.2188**
	19	0	1	52	95 [75; 100]	100 [93; 100]	99 [93; 100]	0.98 [0.93; 1.02]	-	> 0.9999
*Per*-patient	**41**	**6**	**2**	**23**	**95 [84; 99]**	**79 [60; 92]**	**89 [79; 95]**	**0.79 [0.65; 0.93]**	**-**	**0.2891**
	40	2	3	27	93 [81; 99]	93 [77; 99]	93 [85; 98]	0.87 [0.75; 0.98]	-	> 0.9999
T2 Dixon (fat + water)										
Skull	**11**	**1**	**0**	**60**	**100 [72; 100]**	**98 [91; 100]**	**99 [93; 100]**	**0.98 [0.94; 1.02]**	**-**	**> 0.9999**
	11	0	0	61	100 [72; 100]	100 [94; 100]	100 [95; 100]	1.00 [1.00; 1.00]	-	> 0.9999
Thorax	**21**	**4**	**1**	**46**	**95 [77; 100]**	**92 [81; 98]**	**93 [85; 98]**	**0.88 [0.77; 0.98]**	**-**	**0.3750**
	21	2	1	48	95 [77; 100]	96 [86; 100]	96 [88; 99]	0.93 [0.84; 1.01]	-	> 0.9999
Cervical spine	**12**	**1**	**0**	**59**	**100 [74; 100]**	**98 [91; 100]**	**99 [93; 100]**	**0.98 [0.94; 1.02]**	**-**	**> 0.9999**
	12	0	0	60	100 [74; 100]	100 [94; 100]	100 [95; 100]	1.00 [1.00; 1.00]	-	> 0.9999
Thoracic spine	**25**	**7**	**1**	**39**	**96 [80; 100]**	**85 [71; 94]**	**89 [79; 95]**	**0.79 [0.64; 0.93]**	**-**	**0.0703**
	25	3	1	43	96 [80; 100]	93 [82; 99]	94 [86; 98]	0.90 [0.79; 1.00]	-	0.6250
Lumbar spine	**25**	**5**	**2**	**40**	**93 [76; 99]**	**89 [76; 96]**	**90 [81; 96]**	**0.81 [0.68; 0.95]**	**-**	**0.4531**
	29	0	0	43	93 [76; 99]	91 [79; 98]	100 [95; 100]	0.84 [0.72; 0.96]	-	> 0.9999
Pelvis	**38**	**8**	**2**	**24**	**95 [83; 99]**	**75 [57; 89]**	**86 [76; 93]**	**0.73 [0.58; 0.89]**	**-**	**0.1094**
	39	3	1	29	98 [87; 100]	91 [75; 98]	94 [86; 98]	0.89 [0.79; 1.00]	-	0.6250
Humeri	**13**	**3**	**0**	**56**	**100 [75; 100]**	**95 [86; 99]**	**96 [88; 99]**	**0.94 [0.87; 1.01]**	**-**	**0.2500**
	13	0	0	59	100 [75;100]	100 [94; 100]	100 [95; 100]	1.00 [1.00; 1.00]	-	> 0.9999
Femurs	**20**	**4**	**0**	**48**	**100 [83; 100]**	**92 [82; 98]**	**94 [86, 98]**	**0.90 [0.81; 1.00]**	**-**	**0.1250**
	20	0	0	52	100 [83; 100]	100 [93; 100]	100 [95; 100]	1.00 [1.00; 1.00]	-	> 0.9999
*Per*-patient	**41**	**8**	**2**	**21**	**95 [84; 99]**	**72 [53; 87]**	**86 [76; 93]**	**0.74 [0.59; 0.90]**	**-**	**0.1094**
	42	2	1	27	98 [88; 100]	93 [77; 99]	96 [88; 99]	0.92 [0.83; 1.01]	-	> 0.9999

*Notes*: The difference in the proportion of lesions correctly detected in the *per*-region analysis, as well as the difference in the proportion of positive patients correctly detected in the *per*-patient analysis (both compared to the reference standard) is reported with the *p* value of the exact test. A single statistical difference (at *p* < 0.05 but not at *p* < 0.0083 after Bonferroni correction) is observed (*). Entries in boldface: junior reader; not in boldface: senior reader

*TP* true positive, *FP* false positive, *FN* false negative, *TN* true negative, *Se s*ensitivity, *Spe* specificity, *Acc* accuracy, *AC1* Gwet’s AC1 agreement coefficient

In the *per*-patient analysis, two trends were observed. First, the junior reader achieved a similar Se but a lower Sp compared to the senior, regardless of the protocol. Second, the senior reader achieved a slightly higher Se with the T2 Dixon protocol (Se = 98% vs Se = 93% with T1+STIR) while the junior achieved a slightly lower Sp with that protocol (Sp = 72% vs Sp = 79% with T1+STIR).

**Fig. 2:**
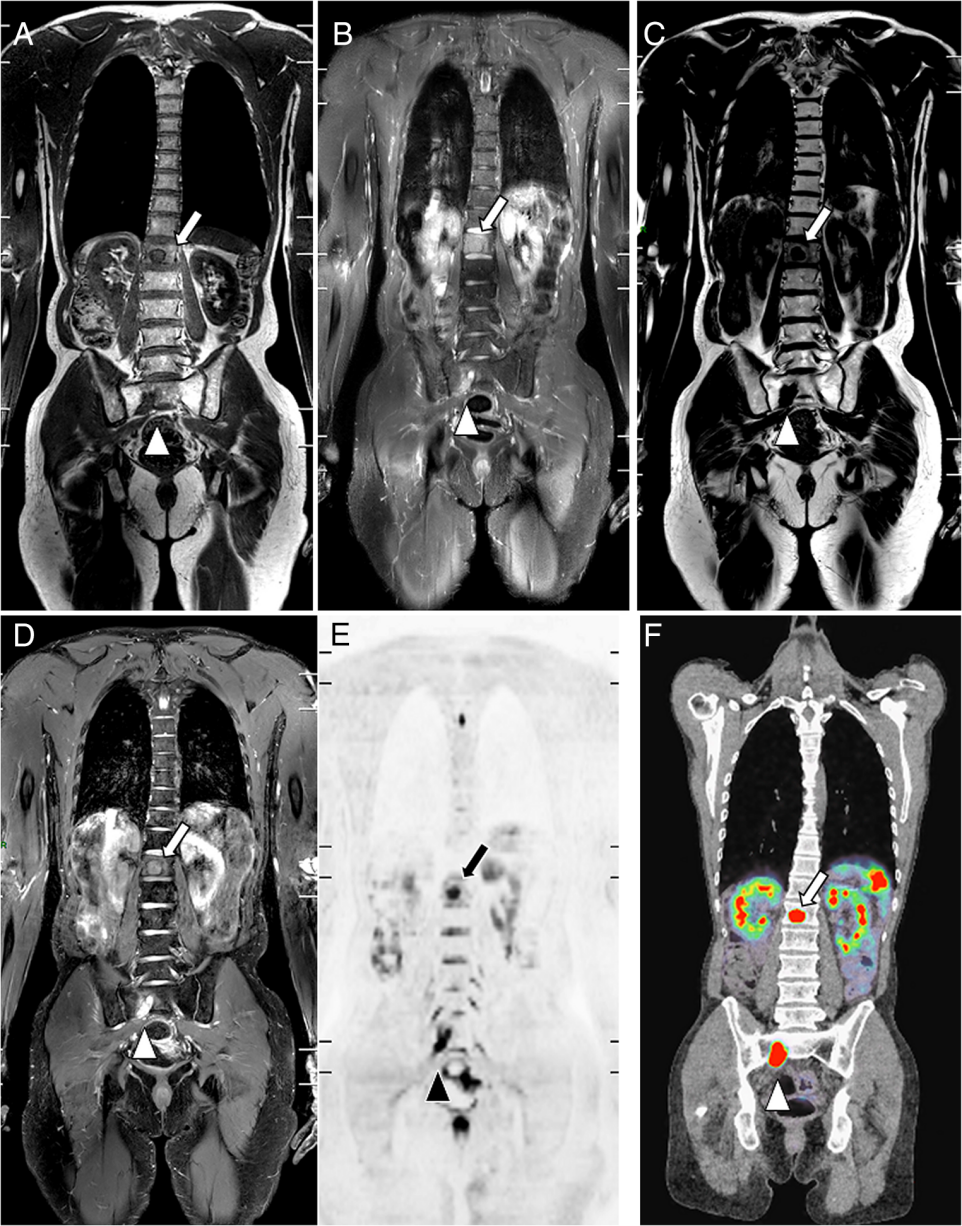
Agreement between sequences on metastatic lesions: WB-MRI study in a 46-year-old man with metastatic neuroendocrine cancer. Two metastases are observed in the L1 vertebral body (arrow) and in the right wing of the sacrum (arrowhead). Coronal T1 (**A**) and STIR (**B**) WB-MRI images show both lesions. Fat (**C**) and water (**D**) reconstructions of the TSE T2 Dixon acquisition show the same lesions. The reference standard, based on reading of all MR images and concurrent imaging studies, confirmed the presence of two metastases. **E** Concurrent DWI image (*b* = 1000 s mm^−2^, inverted gray scale) confirms the presence of both lesions and shows no additional lesion. **F** Gallium-68 dotatate PET/CT fused image shows the same two lesions presenting tracer uptake

**Fig. 3:**
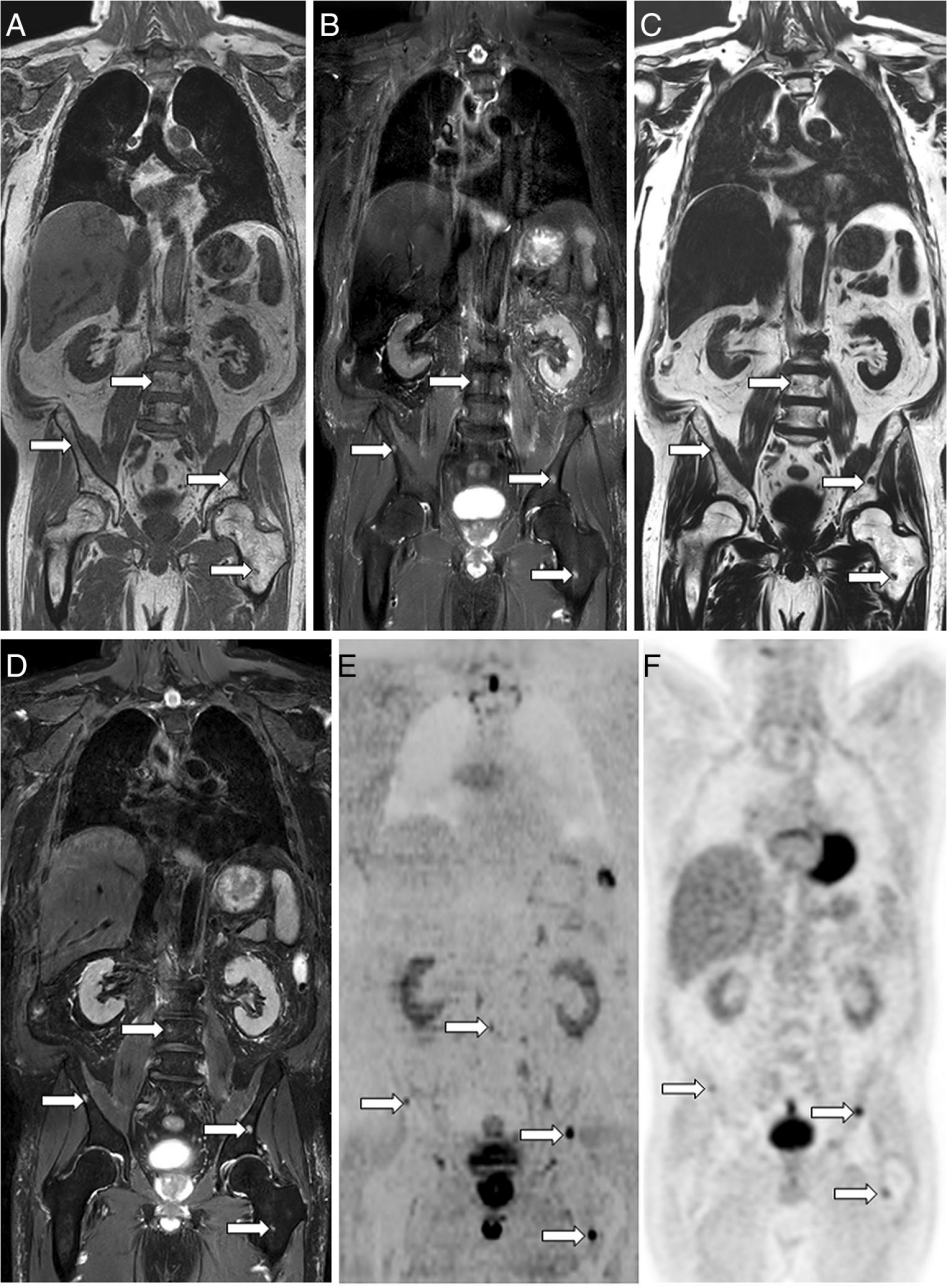
Agreement between sequences on myeloma lesions: WB-MRI study in a 65-year-old man with newly diagnosed multiple myeloma. Several lesions are observed in the lumbar spine, iliac bones, and left femur (arrows). Coronal T1 (**A**) and STIR (**B**) WB-MRI images show four lesions. Fat (**C**) and water (**D**) reconstructions of the TSE T2 Dixon acquisition show the same lesions. The reference standard, based on reading of all MR images and concurrent imaging studies, confirmed the presence of these lesions. **E** Concurrent DWI image (*b* = 1000 s mm^−2^, inverted gray scale) confirms the presence of lesions. **F** [^18^F]F-FDG PET/CT image shows iliac and left femur lesions presenting tracer uptake

Compared to the reference standard, no significant difference was observed in the proportion of lesions correctly detected in the *per*-region analysis, or in the proportion of positive patients correctly detected in the *per*-patient analysis, regardless of both the reader and protocol (Figs. 2 and 3). A non-significant trend suggesting that the junior reader detected more humeral lesions (corresponding to FP) using T1+STIR compared to the reference standard was observed (proportion difference +8.33% [+ 1.28%; + 15.9%], *p* = 0.0313).

In the *per*-patient analysis, the resampling procedure demonstrated a significantly higher Acc of T2 Dixon fat+water compared to T1+STIR when assessed by the senior reader (Acc^Dixon Fat+Water^ = 0.957, Acc^T1+STIR^ = 0.930, mean difference in Acc = +0.027 [+ 0.025; + 0.029], *p* < 0.0001). Conversely, it demonstrated a significantly lower Acc of T2 Dixon fat+water compared to T1+STIR when assessed by the junior (Acc^Dixon Fat+Water^ = 0.860, Acc^T1+STIR^ = 0.889, mean difference in Acc = −0.029 [−0.031; −0.027], *p* < 0.0001).

When assessing differences in Acc according to the patient subgroups, the following observations were made (Supplementary Tables 1 and 2): the senior reader demonstrated a slightly higher Acc using the T2 Dixon protocol in the metastatic patient subgroup only (mean difference in Acc = + 0.036 [+ 0.033; + 0.038], *p* < 0.0001), while the junior reader demonstrated a slightly lower Acc using that protocol, regardless of the patient subgroup (MM: mean difference in Acc = −0.052 [−0.058; −0.046], *p* < 0.0001, metastatic patients: mean difference in Acc= −0.019 [−0.021; −0.017], *p* < 0.0001).

### Semi-quantitative score

In the *per*-patient analysis, agreement between the protocols and the reference standard was good for the junior reader (regardless of the protocol), and very good for the senior reader (regardless of the protocol) (T1+STIR vs reference standard: AC1^junior^ = 0.76 [0.65; 0.87], AC1^senior^ = 0.86 [0.77; 0.95]; T2 Dixon fat+water vs reference standard: AC1^junior^ = 0.78 [0.68; 0.89], AC1^senior^= 0.93 [0.87; 1.00]).

### False-negative and false-positive readings

**Fig. 4:**
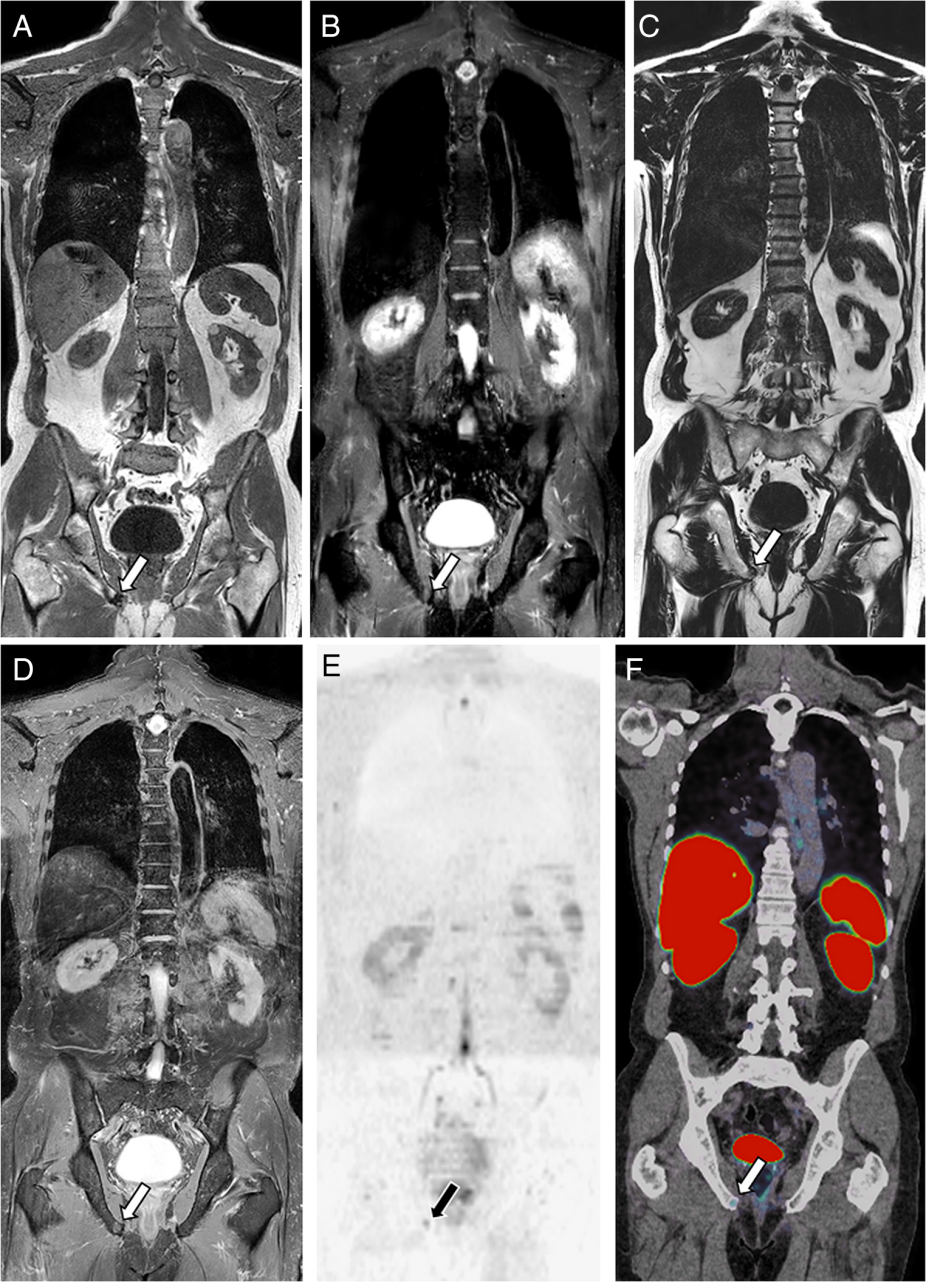
False-negative observation (detection error): WB-MRI in a 71-year-old man with newly diagnosed prostate cancer. Coronal T1 (**A**) and STIR (**B**) WB-MRI images show single centimetric bone metastasis within the right ischio-pubic ramus, with low signal intensity on T1 and high signal intensity on STIR (arrow). The lesion was missed by one observer on these T1 and STIR images. Fat (**C**) and water (**D**) reconstructions of the T2 Dixon acquisition show the same lesion with low signal intensity on the FAT image and high signal intensity on the WATER image (arrows). The reference standard, based on reading of all MR images and concurrent imaging studies, confirmed the presence of a solitary right ischio-pubic lesion. **E** Concurrent DWI image (*b* = 1000 s mm^−2^, inverted gray scale) confirms the presence of the lesion (arrow). **F** Gallium-68 PSMA fused PET/CT image confirms the presence of the lesion

FP readings resulted from benign conditions: degenerative disease (subchondral cysts, juxta-articular/discal changes), enchondromas, focal marrow hyperplasia, vertebral hemangiomas, enostosis, and benign fractures. FN readings resulted from missed lesions (Table 5) (Fig. 4).

**Table 5:** Analysis of the false-positive and false-negative findings, as determined during the consensus adjudication

	Junior reader		Senior reader	
False-positive findings	*N*	Cause	*N*	Cause
Standard (T1+STIR)				
	3	Focal marrow hyperplasia	2	Focal marrow hyperplasia
	7	Degenerative	1	Degenerative
	2	Enchondroma	0	Enchondroma
	3	Angioma	1	Angioma
	1	Enostosis	0	Enostosis
	2	Benign fracture	2	Benign fracture
T2 Dixon fat+water				
	2	Hematopoiesis	2	Hematopoiesis
	6	Degenerative	0	Degenerative
	2	Enchondroma	0	Enchondroma
	4	Angioma	1	Angioma
	2	Enostosis	1	Enostosis
	3	Benign fracture	2	Benign fracture
**False-negative findings**	***N***	**Cause**	***N***	**Cause**
Standard (T1+STIR)				
	3	Thoracic	0	Thoracic
	2	Lumbar	0	Lumbar
	9	Pelvis	3	Pelvis
T2 Dixon fat+water				
	3	Thoracic	3	Thoracic
	2	Lumbar	2	Lumbar
	9	Pelvis	8	Pelvis

False-positive observations are benign conditions considered malignant

False-negative observations are missed lesions; the anatomic location of these false-negative lesions is indicated

## Discussion

This study compared the diagnostic accuracy of a single T2 Dixon sequence including fat+water reconstructions to the guidelines-recommended combination of T1+STIR sequences used as morphological sequences in WB-MRI examinations performed to detect skeletal metastases or MM lesions [7–9]. Our results showed that the accuracy (Acc) of the combination of T2 Dixon fat+water reconstructions was similar (−2.9%; +2.7%) to that of the reference protocol combining T1+STIR sequences.

These results are in line with published studies that compared the performance of a T2 Dixon sequence and of the combination of T1 and fat-suppressed fluid-sensitive sequences in MRI studies limited to the spine or to a spine segment for the detection of metastases or MM [26–28]. These three studies concluded that the T2 Dixon protocol had similar diagnostic performance compared to the standard protocol, providing STIR-like (fluid sensitive) information with the water image, and T1-like (fat sensitive) information with the fat image, and that its use significantly reduces the acquisition time of spine MRI in oncologic indications.

The present study extends this conclusion to WB-MRI examinations used for skeletal screening.

Both repeatability and reproducibility of readings were at least good for both the T2 Dixon and the T1+STIR protocols, for both the categorical (disease present/absent) and semi-quantitative scores (lesion count), and in both the regional and global (*per*-patient) analyses. Of note, all quantitative evaluations of repeatability/reproducibility (Gwet’s coefficient) were higher for the T2 Dixon evaluation compared to T1+STIR.

Regarding image quality, the T1 sequence had higher SNR compared to the T2 Dixon fat images in lesions and reference areas. This difference is not unexpected as the T1 sequence is acquired in 3D mode, which intrinsically provides a higher signal than 2D sequences [42]. This differs from the studies of Maeder and Danner, where a higher SNR for the T2 Dixon fat images compared to the T1 images acquired in 2D mode was found [26, 28]. In our study, the T1 and the T2 Dixon fat sequences had similar CNR. Interestingly, T2 Dixon fat images had significantly higher CRR compared to T1 images, and T2 Dixon water images had significantly higher CRR compared to STIR images. In practice, this higher CRR results in an improved detectability of the lesions.

Regarding the diagnostic performance, both protocols showed similar levels of sensitivity and specificity in the *per*-region analysis, with no significant difference between them in the proportion of correctly detected lesions, compared to the reference standard. Some FP and FN findings were observed, presenting similar causes for both protocols and both readers.

The junior reader had a higher number of FP observations compared to the senior, resulting in a lower specificity and accuracy, in both the *per*-region and *per*-patient analyses. FP observations were errors of interpretation of degenerative changes or benign conditions, already reported as pitfalls in previous studies of the diagnostic performance of WB-MRI for the detection of bone marrow lesions [11, 43]. These causes of FP should be taken into account during the learning phase of less-experienced readers to avoid overdiagnosis.

Both readers had a low number of FN observations, leading to high sensitivity of both the T2 Dixon and T1+STIR protocols. FNs were due to errors of detection, i.e. lesions missed by the reader or lesions not seen on the available sequences but detected by the reference standard.

Considering the high level of accuracy achieved in this study and the shorter acquisition time, the T2 Dixon protocol represents a realistic alternative to the classical T1+STIR combination for the skeletal screening and follow-up of patients with metastases from solid cancers and MM. In our center, its implementation allows sparing 9 min 12s in the acquisition time of morphological sequences. The total acquisition time of WB-MRI including T2 Dixon and DWI sequences is 27 min 03 s.

This study has several limitations. First, it was performed in a single center and on a single MRI magnet. However, the cohort of patients was large and representative of current WB-MRI indications. Enlarging the cohort of patients should allow refining the evaluation of the difference in accuracy between protocols. Extending our protocol in a multi-centric study including patients imaged on different MRI magnets and multiple readings should allow the generalization of our observations.

Second, only the fat and water reconstructions derived from the T2 Dixon sequence were used, excluding IP and OP images. This choice relies on previous observations showing the lack of added value of IP and OP images to detect bone lesions [26]. However, the availability of IP and OP images may be an additional advantage of the T2 Dixon protocol. Indeed, IP images are equivalent to non-fat-suppressed T2-weighted images and provide anatomic information that may be useful in the evaluation of the spinal canal and spinal cord, in the characterization of vertebral fractures, and in the detection of visceral and lymph node metastases without additional imaging time [44]. Moreover, the availability of IP and OP images and their comparisons may help in the determination of the neoplastic nature of an ambiguous bone marrow lesion and in the recognition of focal marrow hyperplasia and acute benign vertebral fractures, which are frequent pitfalls causing FP observations. Benign lesions indeed show a signal dropout on OP images due to the intravoxel coexistence of microscopic fatty components and hydrated normal cells within the bone marrow [45, 46]. Besides this qualitative approach, the T2 Dixon sequence allows fat fraction (FF) quantification, which can be used to differentiate benign and malignant lesions [24]. Further studies should assess the added value of IP and OP images and of FF measurements.

Third, this study only assessed the diagnostic performance of morphological sequences, in a time-saving perspective. Although the DWI sequence was systematically available, we did not compare its diagnostic accuracy to that of anatomic sequences. The high accuracy (close to 1.00) of the T2 Dixon fat+water protocol suggests that this morphologic sequence alone may be sufficient for skeletal screening, with no need for DWI. This high performance of the anatomic T2 Dixon for bone screening, making DWI superfluous in this indication, has been suggested in a study in 5 patients with bone metastases of renal cancer [47]. We did not question the recommended systematic combination of anatomic and functional DWI sequences. Indeed, DWI sequences allow optimization of bone lesion detection, screening of lymph nodes and visceral lesions, and provide ADC measurements useful for lesion characterization and assessment of treatment response [7, 9, 48–50].

## Conclusion

This study in patients with bone metastases or MM shows that a shorter anatomical WB-MRI protocol relying on a single T2 Dixon sequence with fat and water reconstructions may replace the reference T1+STIR sequences for skeletal screening, shortening examination duration, without loss of diagnostic accuracy.

## Supplementary Information

ESM 1 (PDF 215 kb)

## Abbreviations

AC1: Gwet’s agreement coefficient
Acc: Accuracy
BVC: Best valuable comparator
CNR: Contrast-to-noise ratio
CRR: Contrast-to-reference ratio
CT TAP: Computed tomography of thorax, abdomen, and pelvis
DWI: Diffusion-weighted imaging
FF: Fat fraction
FN: False negative
FP: False positive
IP: In-phase imaging from the T2 Dixon sequence
MM: Multiple myeloma
OP: Out-of-phase imaging from the T2 Dixon sequence
PET: Positron emission tomography
ROI: Region of interest
Se: Sensitivity
SI: Signal intensity
SNR: Signal-to-noise ratio
Sp: Specificity
STIR: Short-Tau inversion recovery
TN: True negative
TP: True positive
TSE: Turbo spin echo
WB-MRI: Whole-body magnetic resonance imaging

## Declaration

### Guarantor

The scientific guarantor of this publication is Frederic E. Lecouvet.

### Statistics and biometry

One of the authors (Nicolas Michoux) has significant statistical expertise.

### Methodology

• retrospective

• cross-sectional study

• performed at one institution
