# Supplementary material for: Whole-body MRI in oncology: can a single anatomic T2 Dixon sequence replace the combination of T1 and STIR sequences to detect skeletal metastasis and myeloma?
Source: Eur Radiol. 2022 Aug 4;33(1):244–57. doi: 10.1007/s00330-022-09007-8 (PMC9755082; doi:10.1007/s00330-022-09007-8)
Supplement: Supplementary file 1 — (PDF 215 kb) [file 330_2022_9007_MOESM1_ESM.pdf]

Supplementary Materials

Results

Diagnostic characteristics in the subgroup of patients with MM (Supplementary Table 1)

In the *per*-region analysis, Se of T1+STIR was ≥89% for the senior reader (≥100% for the junior) regardless of the region. Sp was ≥91% for the senior reader (≥73% for the junior) regardless of the region. Se of T2 Dixon Fat+Water was =100% regardless of both the reader and the region. Sp was ≥82% for the senior reader (≥64% for the junior).

In the *per*-patient analysis, the junior reader achieved a lower Sp compared to the senior, regardless of the protocol. The senior reader achieved a slightly higher Se with the T2 Dixon protocol (at the cost of a slightly lower Sp) while the junior achieved a slightly lower Sp with that protocol (without change in Se).

Compared to the reference standard, no significant difference was observed in the proportion of lesions correctly detected in the *per*-region analysis, or in the proportion of positive patients correctly detected in the *per*-patient analysis, regardless of both the reader and protocol (all p-values > 0.05).

Diagnostic characteristics in the subgroup of patients with solid cancers at high risk for metastases (Supplementary Table2)

In the *per*-region analysis, Se of T1+STIR was ≥92% for the senior reader (≥86% for the junior) regardless of the region. Sp was ≥95% for the senior reader (≥84% for the junior) regardless of the region. Se of T2 Dixon Fat+Water was ≥90% regardless of both the reader and the region. Sp was ≥94% for the senior reader (≥81% for the junior).

In the *per*-patient analysis, the junior reader achieved a similar Se but a lower Sp compared to the senior, regardless of the protocol. The senior reader achieved a slightly higher Sp with the T2 Dixon protocol while the junior achieved a slightly lower Sp with that protocol.

Compared to the reference standard, no significant difference was observed in the proportion of lesions correctly detected in the *per*-region analysis, or in the proportion of positive patients correctly detected in the *per*-patient analysis, regardless of both the reader and protocol (all p-values > 0.05).

Difference of performance according to the patients subgroup

With the senior reader, the resampling procedure demonstrated no significant difference in Acc between both protocols in the MM subgroup ($Acc^{Dixon\ Fat+Water}$=0.898, $Acc^{T1+STIR}$=0.892, p=0.0164), and a slightly higher Acc of the T2 Dixon protocol in the metastatic patients subgroup ($Acc^{Dixon\ Fat+Water}$=0.980, $Acc^{T1+STIR}$=0.945, mean difference in Acc=+0.036 [+0.033;+0.038], p<0.0001). These differences in Acc were due to a slightly higher Se when using the T2 Dixon protocol, compensated by a slightly lower Sp in the MM patients subgroup, while also accompanied with a slightly higher Sp in the metastatic patients subgroup.

With the junior reader, the resampling procedure demonstrated a slightly lower Acc of the T2 Dixon protocol in the MM subgroup ($Acc^{Dixon\ Fat+Water}$=0.846, $Acc^{T1+STIR}$=0.898, mean difference in Acc=-0.052 [-0.058;-0.046], p<0.0001), and also a slightly lower Acc of the T2 Dixon protocol in the metastatic patients subgroup ($Acc^{Dixon\ Fat+Water}$=0.865, $Acc^{T1+STIR}$=0.884, mean difference in Acc=-0.019 [-0.021;-0.017], p<0.0001). These differences in Acc were due to an absence of change in Se while a slightly lower Sp was achieved in both subgroups of patients when using the T2 Dixon protocol.

Tables

Supplementary Table 1. Diagnostic characteristics and agreement between the protocols and the Reference Standard in the group of patients with Multiple Myeloma (N=20). The difference in proportion of lesions correctly detected in the *per*-region-analysis, as well as the difference in proportion of positive patients correctly detected in the *per*-patient analysis (both compared to the Reference Standard) is reported with the *p*-value of the Exact test. White line: junior reader, Grey line: senior reader.

**Standard (3D T1 + T2 STIR)**										
	**TP**	**FP**	**FN**	**TN**	**Se**	**Sp**	**Acc**	**AC1**	**Proportion difference (in %)**	***p*-value**
Skull	6	1	0	13	100 [54; 100]	93 [66; 100]	95 [75; 100]	0.91 [0.74; 1.08]	-	> 0.9999
	6	0	0	14	100 [54; 100]	100 [77; 100]	100 [83; 100]	1.00 [1.00; 1.00]	-	> 0.9999
Thorax	8	1	0	11	100 [63; 100]	92 [62; 100]	95 [75; 100]	0.90 [0.71; 1.09]	-	> 0.9999
	8	0	0	12	100 [63; 100]	100 [74; 100]	100 [83; 100]	1.00 [1.00; 1.00]	-	> 0.9999
Cervical spine	6	1	0	13	100 [54; 100]	93 [66; 100]	95 [75; 100]	0.91 [0.74; 1.08]	-	> 0.9999
	6	0	0	14	100 [54; 100]	100 [77; 100]	100 [83; 100]	1.00 [1.00; 1.00]	-	> 0.9999
Thoracic spine	9	1	0	10	100 [66; 100]	91 [59; 100]	95 [75; 100]	0.90 [0.71; 1.09]	-	> 0.9999
	9	1	0	10	100 [66; 100]	91 [59; 100]	95 [75; 100]	0.90 [0.71; 1.09]	-	> 0.9999
Lumbar spine	6	3	0	11	100 [54; 100]	79 [49; 95]	85 [62; 97]	0.71 [0.41; 1.02]	-	0.2500
	6	1	0	13	100 [54; 100]	93 [66; 100]	95 [75; 100]	0.91 [0.74; 1.08]	-	> 0.9999
Pelvis	9	3	0	8	100 [66; 100]	73 [39; 94]	85 [62; 97]	0.70 [0.39; 1.01]	-	0.2500
	8	1	1	10	89 [52; 100]	91 [59; 100]	90 [68; 99]	0.80 [0.54; 1.06]	-	> 0.9999
Humeri	6	3	0	11	100 [54; 100]	79 [49; 95]	85 [62; 97]	0.71 [0.41; 1.02]	-	0.2500
	6	0	0	14	100 [54; 100]	100 [77; 100]	100 [83; 100]	1.00 [1.00; 1.00]	-	> 0.9999
Femurs	7	3	0	10	100 [59; 100]	77 [46; 95]	85 [62; 97]	0.71 [0.40; 1.02]	-	0.2500
	7	0	0	13	100 [59; 100]	100 [75; 100]	100 [83; 100]	1.00 [1.00; 1.00]	-	> 0.9999
per-Patient	11	2	0	7	100 [72; 100]	78 [40; 97]	90 [68; 99]	0.81 [0.55; 1.06]	-	0.5000
	10	1	1	8	91 [59; 100]	89 [52; 100]	90 [68; 99]	0.81 [0.54; 1.06]	-	> 0.9999
**T2 Dixon (Fat + Water)**										
	**TP**	**FP**	**FN**	**TN**	**Se**	**Sp**	**Acc**	**AC1**	**Proportion difference (in %)**	***p*-value**
Skull	6	1	0	13	100 [54; 100]	93 [66; 100]	95 [75; 100]	0.91 [0.74; 1.08]	-	> 0.9999
	6	0	0	14	100 [54; 100]	100 [77; 100]	100 [83; 100]	1.00 [1.00; 1.00]	-	> 0.9999
Thorax	8	1	0	11	100 [63; 100]	92 [62; 100]	95 [75; 100]	0.90 [0.71; 1.09]	-	> 0.9999
	8	0	0	12	100 [63; 100]	100 [74; 100]	100 [83; 100]	1.00 [1.00; 1.00]		> 0.9999
Cervical spine	6	1	0	13	100 [54; 100]	93 [66; 100]	95 [75; 100]	0.91 [0.74; 1.08]	-	> 0.9999
	6	0	0	14	100 [54; 100]	100 [77; 100]	100 [83; 100]	1.00 [1.00; 1.00]		> 0.9999
Thoracic spine	9	2	0	9	100 [66; 100]	82 [48; 98]	90 [68; 99]	0.80 [0.54; 1.06]	-	0.5000
	9	2	0	9	100 [66; 100]	82 [48; 98]	90 [68; 99]	0.80 [0.54; 1.06]		0.5000
Lumbar spine	6	2	0	12	100 [54; 100]	86 [57; 98]	90 [68; 99]	0.82 [0.57; 1.07]	-	0.5000
	6	2	0	12	100 [54; 100]	86 [57; 98]	90 [68; 99]	0.82 [0.57; 1.07]		0.5000
Pelvis	9	4	0	7	100 [66; 100]	64 [31; 89]	80 [56; 94]	0.60 [0.25; 0.95]	-	0.1250
	9	2	0	9	100 [66; 100]	82 [48; 98]	90 [68; 99]	0.80 [0.54; 1.06]		0.5000
Humeri	6	1	0	13	100 [54; 100]	93 [66; 100]	95 [75; 100]	0.91 [0.74; 1.08]	-	> 0.9999
	6	0	0	14	100 [54; 100]	100 [77; 100]	100 [83; 100]	1.00 [1.00; 1.00]		> 0.9999
Femurs	7	1	0	12	100 [59; 100]	92 [64; 100]	95 [75; 100]	0.91 [0.72; 1.09]	-	> 0.9999
	7	0	0	13	100 [59; 100]	100 [75; 100]	100 [83; 100]	1.00 [1.00; 1.00]		> 0.9999
per-Patient	11	3	0	6	100 [72; 100]	67 [30; 93]	85 [62; 97]	0.72 [0.41; 1.02]	-	0.2500
	11	2	0	7	100 [72; 100]	78 [40; 97]	90 [68; 99]	0.81 [0.55; 1.06]	-	0.5000

Notes: The difference in proportion of lesions correctly detected in the per-region-analysis, as well as the difference in proportion of positive patients correctly detected in the per-patient analysis (both compared to the Reference Standard) is reported with the p-value of the Exact test. A single statistical difference (at $p<0.05$ but not at $p<0.0083$ after Bonferroni correction) is observed. White line: junior reader, Grey line: senior reader.
TP: true positive; FP: false positive ; FN: false negative; TN: true negative; Se: Sensitivity; Spe: Specificity; Acc: Accuracy; AC1: Gwet's AC1 agreement coefficient.

Supplementary Table 2. Diagnostic characteristics and agreement between the protocols and the Reference Standard in the group of patients with metastases (N=52). The difference in proportion of lesions correctly detected in the *per*-region-analysis, as well as the difference in proportion of positive patients correctly detected in the *per*-patient analysis (both compared to the Reference Standard) is reported with the *p*-value of the Exact test. White line: junior reader, Grey line: senior reader.

	TP	FP	FN	TN	Se	Sp	Acc	AC1	Proportion difference (in %)	*p*-value
**Standard (3D T1 + T2 STIR)**										
Skull	5	0	0	47	100 [48; 100]	100 [93; 100]	100 [93; 100]	1.00 [1.00; 1.00]	-	> 0.9999
	5	0	0	47	100 [48; 100]	100 [93; 100]	100 [93; 100]	1.00 [1.00; 1.00]	-	> 0.9999
Thorax	13	4	1	34	93 [66; 100]	89 [75; 97]	90 [79; 97]	0.83 [0.69; 0.98]	-	0.3750
	13	1	1	37	93 [66; 100]	97 [86; 100]	96 [87; 100]	0.94 [0.85; 1.02]	-	> 0.9999
Cervical spine	6	3	0	43	100 [54; 100]	93 [82; 99]	94 [84; 99]	0.92 [0.83; 1.01]	-	0.2500
	6	0	0	46	100 [54; 100]	100 [92; 100]	100 [93; 100]	1.00 [1.00; 1.00]	-	> 0.9999
Thoracic spine	16	4	1	31	94 [71; 100]	89 [73; 97]	90 [79; 97]	0.82 [0.67; 0.97]	-	0.3750
	17	0	0	35	100 [81; 100]	100 [90; 100]	100 [93; 100]	1.00 [1.00; 1.00]	-	> 0.9999
Lumbar spine	18	5	3	26	86 [64; 97]	84 [66; 95]	85 [72; 93]	0.70 [0.50; 0.89]	-	0.7266
	20	0	1	31	95 [76; 100]	100 [89; 100]	98 [90; 100]	0.96 [0.89; 1.03]	-	> 0.9999
Pelvis	29	2	2	19	94 [79; 99]	90 [70; 99]	92 [81; 98]	0.85 [0.71; 0.99]	-	> 0.9999
	29	1	2	20	94 [79; 99]	95 [76; 100]	94 [84; 99]	0.89[0.76; 1.01]	-	> 0.9999
Humeri	7	3	0	42	100 [59; 100]	93 [82; 99]	94 [84; 99]	0.92 [0.83; 1.01]	-	0.2500
	7	2	0	43	100 [59; 100]	96 [85; 100]	96 [87; 100]	0.95 [0.87; 1.02]	-	0.5000
Femurs	12	2	1	37	92 [64; 100]	95 [83; 99]	94 [84; 99]	0.91 [0.80; 1.01]	-	> 0.9999
	12	0	1	39	92 [64; 100]	100 [91; 100]	98 [90; 100]	0.97 [0.91; 1.03]	-	> 0.9999
per-Patient	30	4	2	16	94 [79; 99]	80 [56; 94]	88 [77; 96]	0.78 [0.62; 0.95]	-	0.6875
	30	1	2	19	94 [79; 99]	95 [75; 100]	94 [84; 99]	0.89 [0.77; 1.01]	-	> 0.9999
**T2 Dixon (Fat + Water)**										
	**TP**	**FP**	**FN**	**TN**	**Se**	**Sp**	**Acc**	**AC1**	**Proportion difference (in %)**	***p*-value**
Skull	5	0	0	47	100 [48; 100]	100 [93; 100]	100 [93; 100]	1.00 [1.00; 1.00]	-	> 0.9999
	5	0	0	47	100 [48; 100]	100 [93; 100]	100 [93; 100]	1.00 [1.00; 1.00]	-	> 0.9999
Thorax	13	3	1	35	93 [66; 100]	92 [79; 98]	92 [81; 98]	0.87 [0.74; 1.00]	-	0.6250
	13	2	1	36	93 [66; 100]	95 [82; 99]	94 [84; 99]	0.90 [0.79; 1.01]	-	> 0.9999
Cervical spine	6	0	0	46	100 [54; 100]	100 [92; 100]	100 [93; 100]	1.00 [1.00; 1.00]	-	> 0.9999
	6	0	0	46	100 [54; 100]	100 [92; 100]	100 [93; 100]	1.00 [1.00; 1.00]	-	> 0.9999
Thoracic spine	16	5	1	30	94 [71; 100]	86 [70; 95]	88 [77; 96]	0.78 [0.62; 0.95]	-	0.2188
	16	1	1	34	94 [71; 100]	97 [85; 100]	96 [87; 100]	0.93 [0.84; 1.03]	-	> 0.9999
Lumbar spine	19	3	2	28	90 [70; 99]	90 [74; 98]	90 [79; 97]	0.81 [0.66; 0.97]	-	> 0.9999
	19	2	2	29	90 [70; 99]	94 [79; 99]	92 [81; 98]	0.85 [0.71; 0.99]	-	> 0.9999
Pelvis	29	4	2	17	94 [79; 99]	81 [58; 95]	88 [77; 96]	0.78 [0.61; 0.95]	-	0.6875
	30	1	1	20	97 [83; 100]	95 [76; 100]	96 [87; 100]	0.93 [0.82; 1.03]	-	> 0.9999
Humeri	7	2	0	43	100 [59; 100]	100 [92; 100]	96 [87; 100]	0.95 [0.87; 1.02]	-	0.5000
	7	0	0	45	100 [59; 100]	100 [92; 100]	100 [93; 100]	1.00 [1.00; 1.00]	-	> 0.9999
Femurs	13	3	0	36	100 [75; 100]	92 [79; 98]	94 [84; 99]	0.90 [0.79; 1.01]	-	0.2500
	13	0	0	39	100 [75; 100]	100 [91; 100]	100 [93; 100]	1.00 [1.00; 1.00]	-	> 0.9999
per-Patient	30	5	2	15	94 [79; 99]	75 [51; 91]	87 [74; 94]	0.75 [0.57; 0.93]	-	0.4531
	31	0	1	20	97 [84; 100]	100 [83; 100]	98 [90; 100]	0.96 [0.89; 1.03]	-	> 0.9999

Notes: The difference in proportion of lesions correctly detected in the per-region-analysis, as well as the difference in proportion of positive patients correctly detected in the per-patient analysis (both compared to the Reference Standard) is reported with the p-value of the Exact test. A single statistical difference (at p<0.05 but not at p<0.0083 after Bonferroni correction) is observed. White line: junior reader, Grey line: senior reader.
TP: true positive; FP: false positive ; FN: false negative; TN: true negative; Se: Sensitivity; Spe: Specificity; Acc: Accuracy; AC1: Gwet's AC1 agreement coefficient.
